# Supplementary figures and images for: The Yeast and Hypha Phases of Candida krusei Induce the Apoptosis of Bovine Mammary Epithelial Cells via Distinct Signaling Pathways
Source: Animals (Basel). 2023 Oct 15;13(20):3222. doi: 10.3390/ani13203222 (PMC10603689; doi:10.3390/ani13203222)

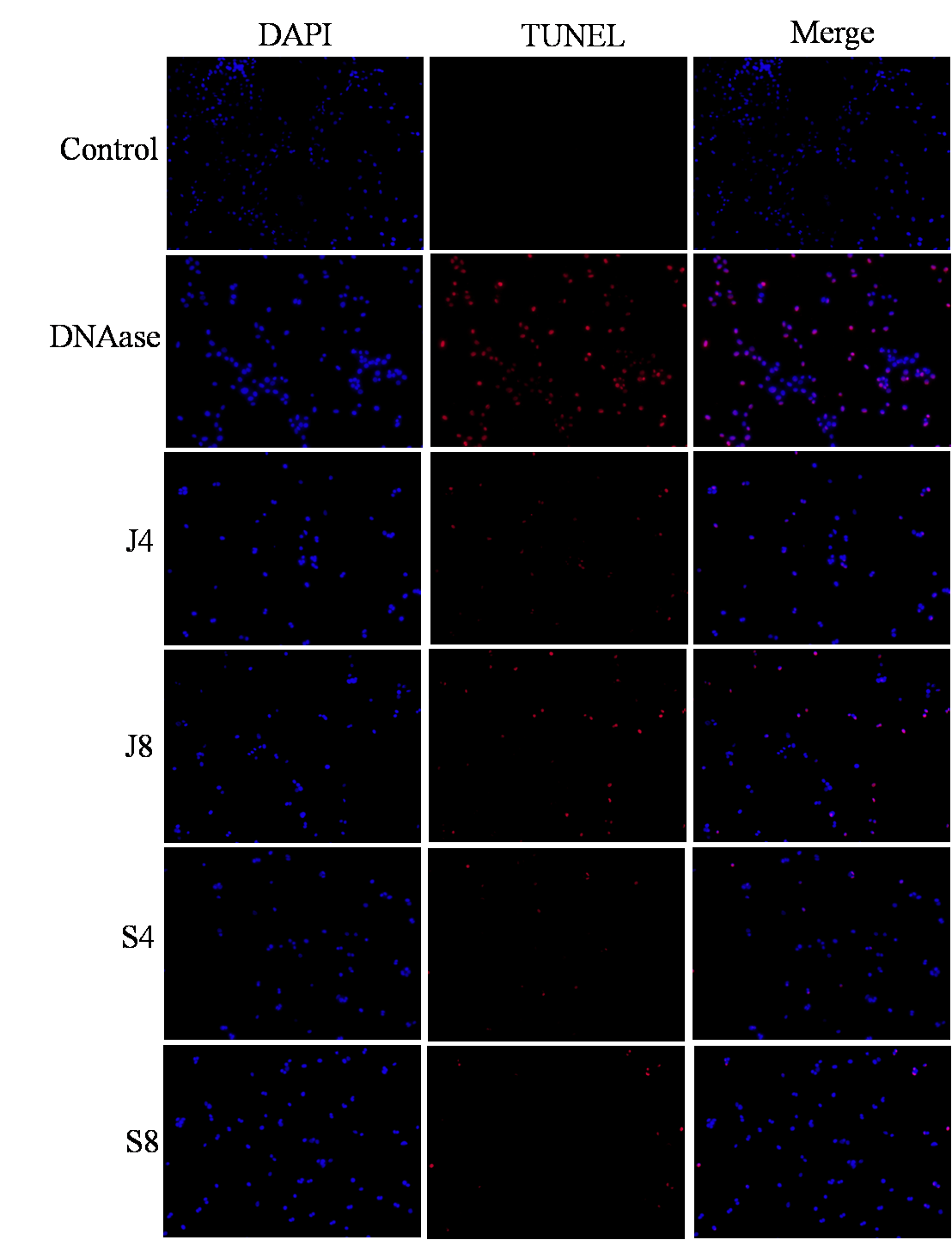

Supplement: Supplementary file 1 [file animals-13-03222-s001.zip › Supplemental Figure S1.tif]

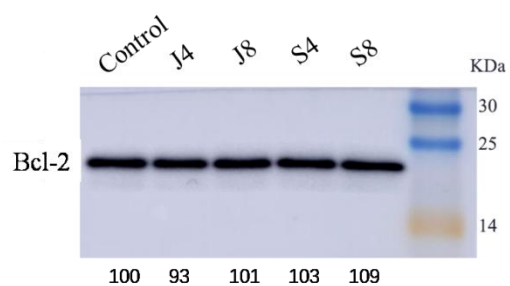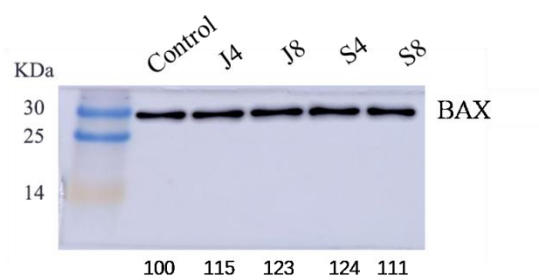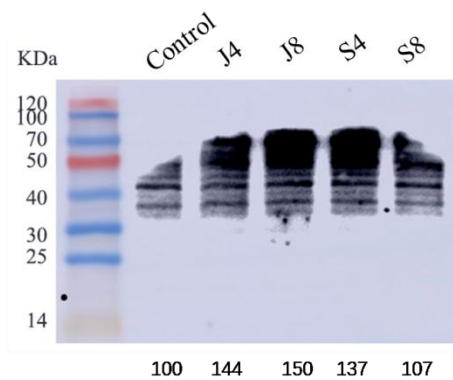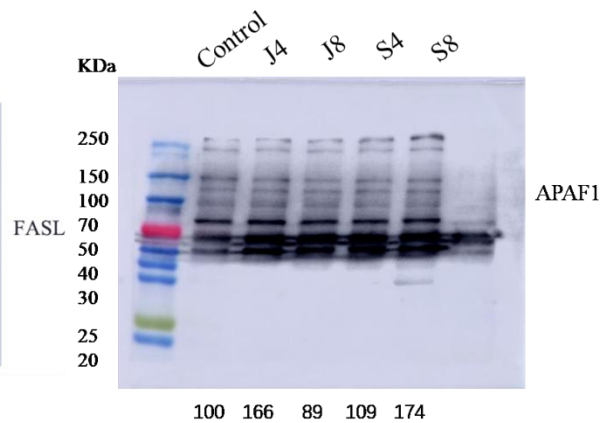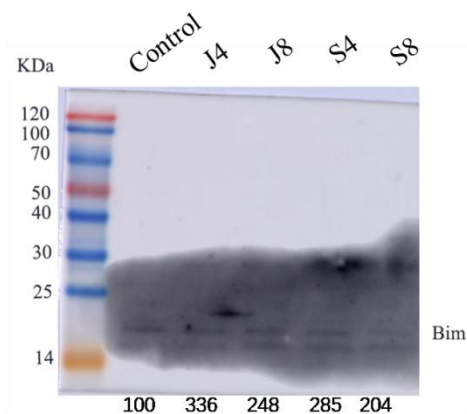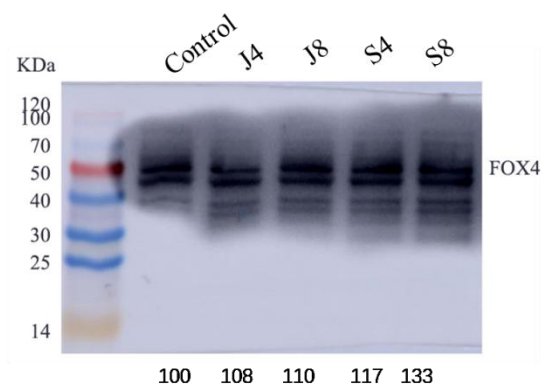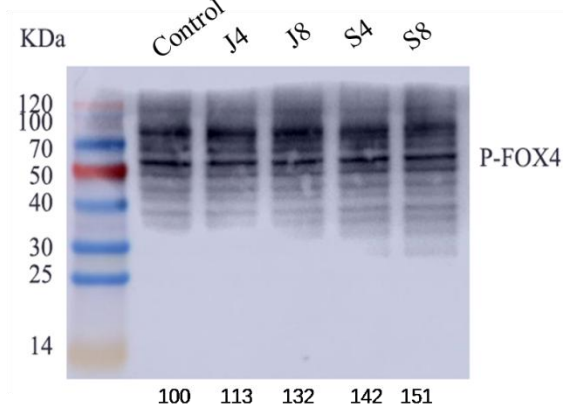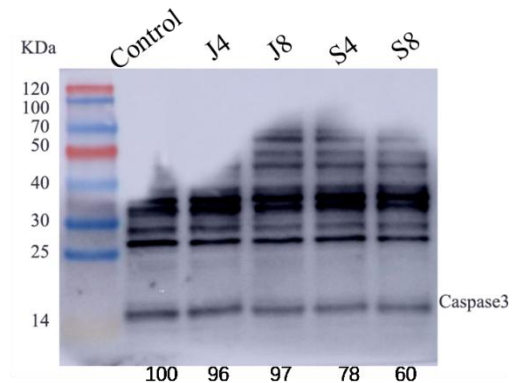

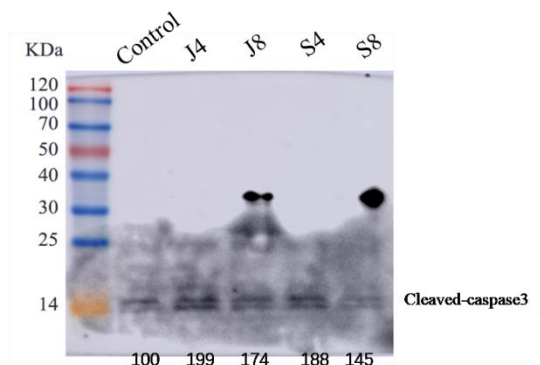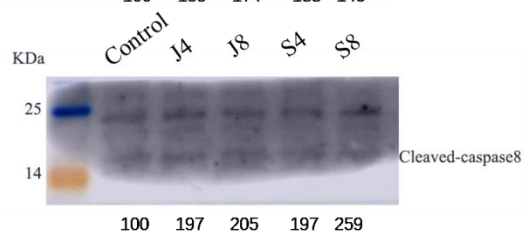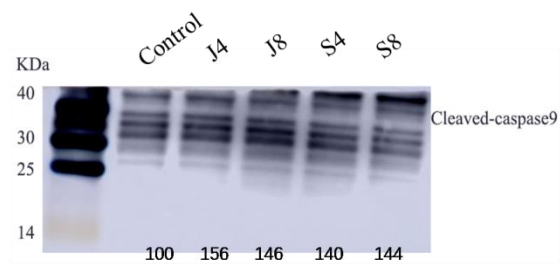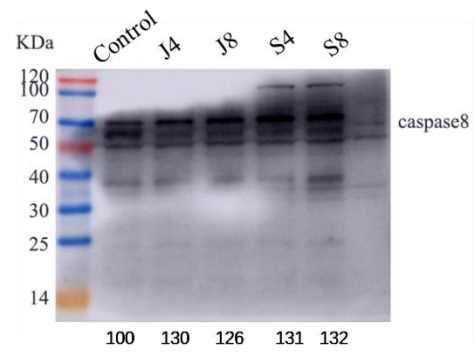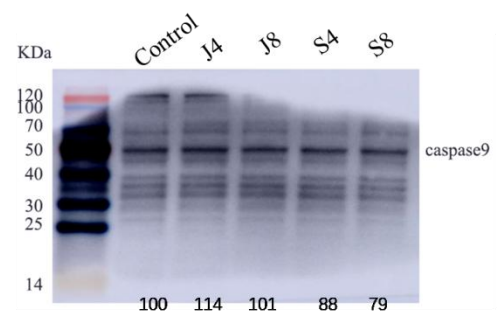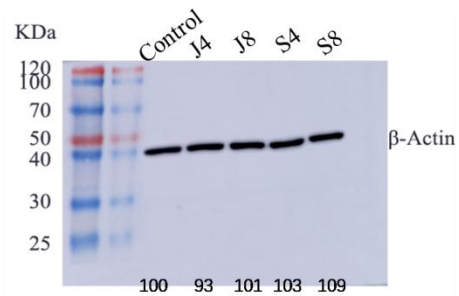

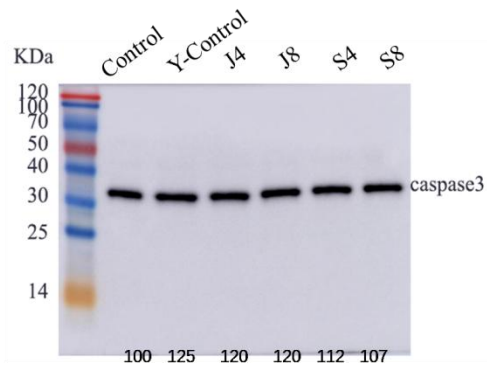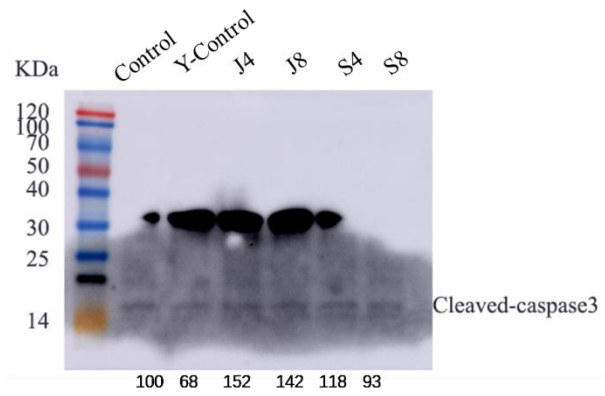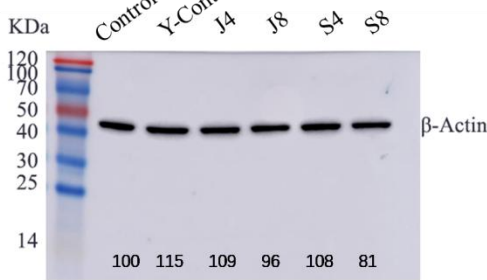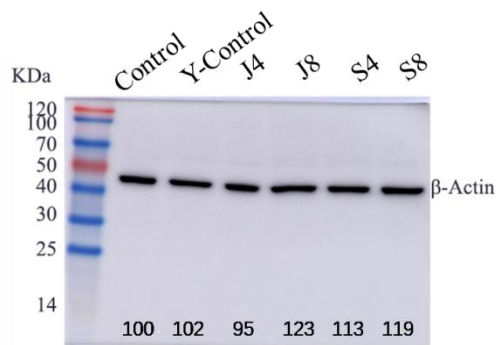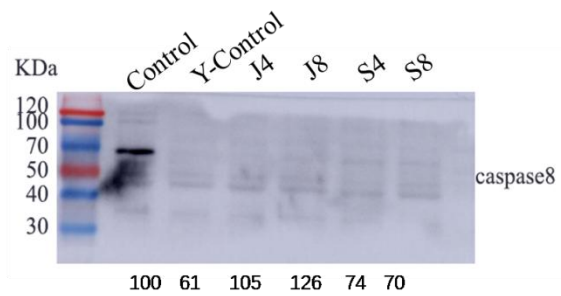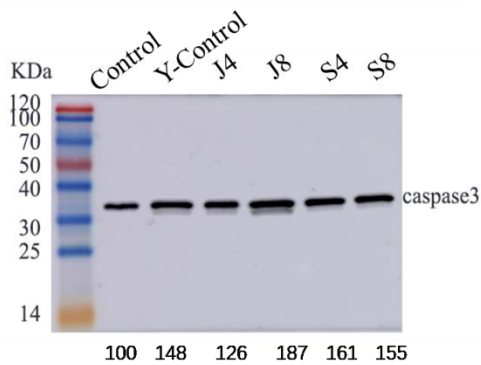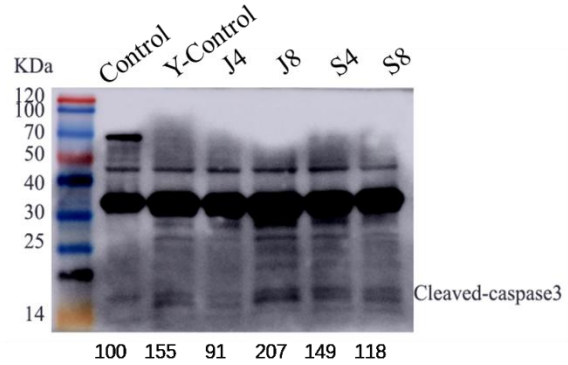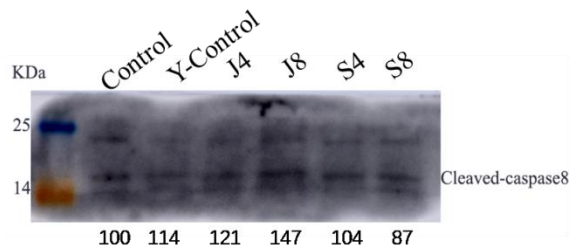

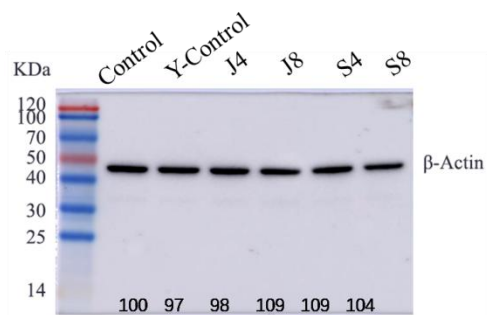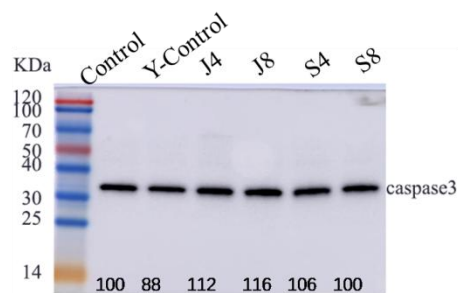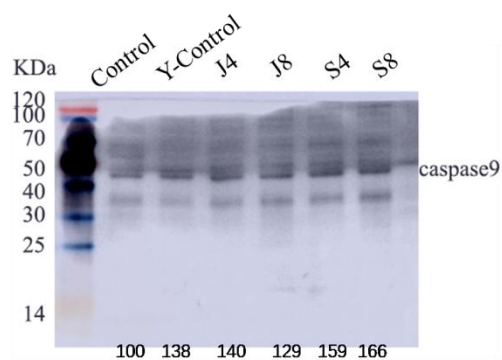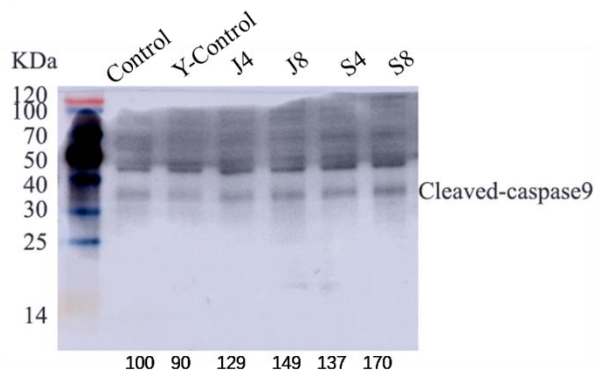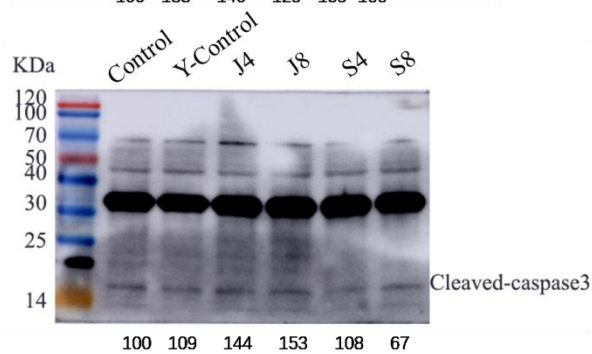

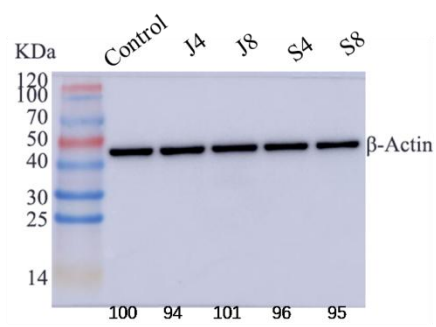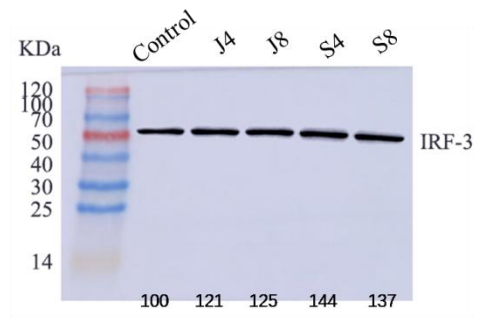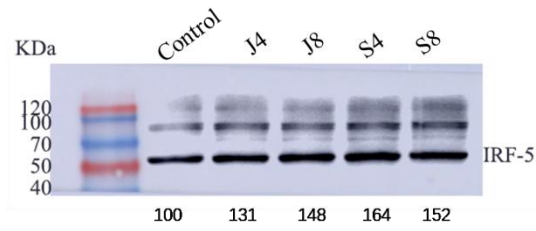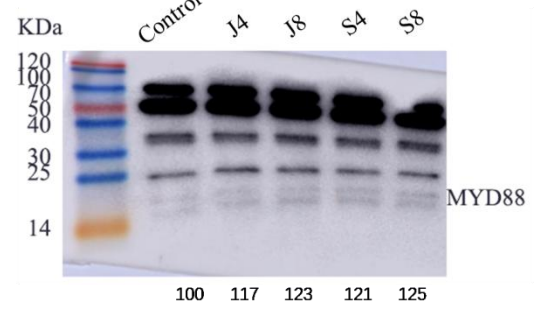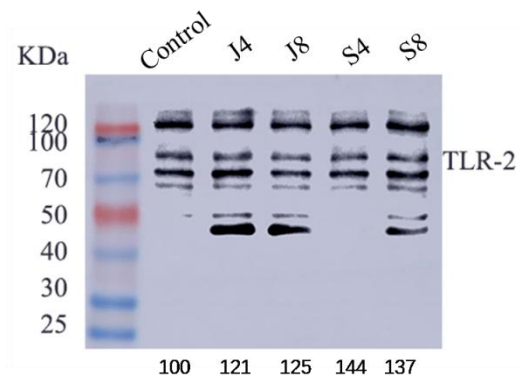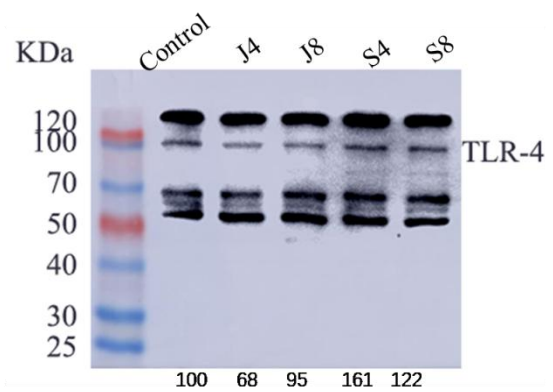

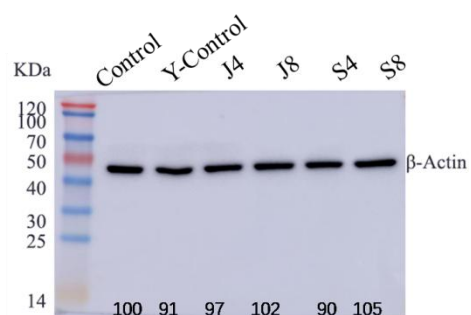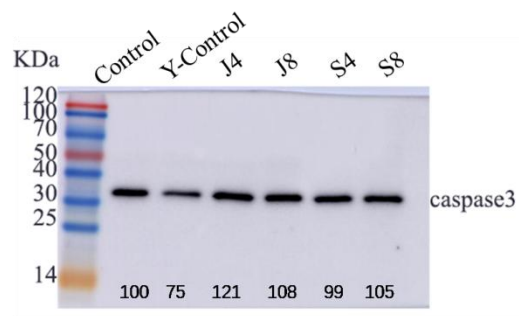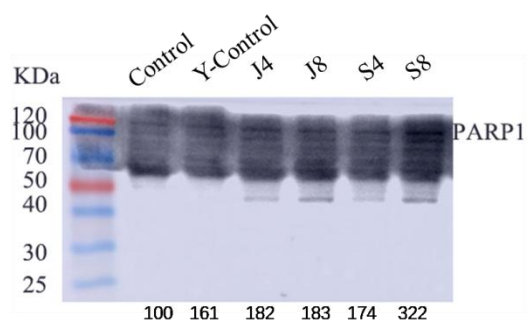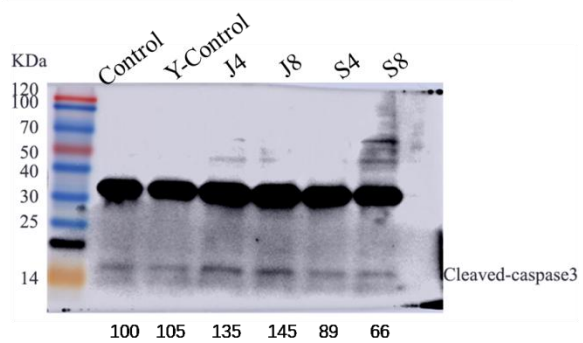

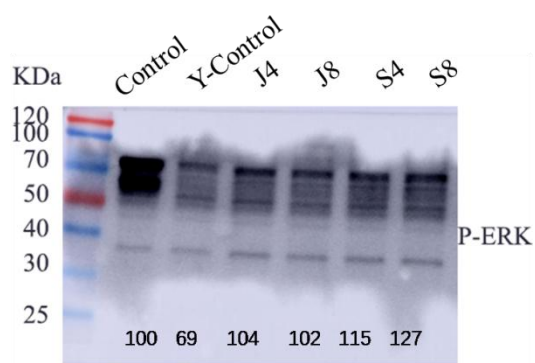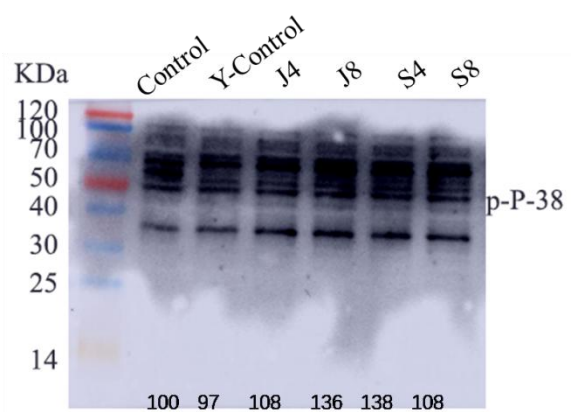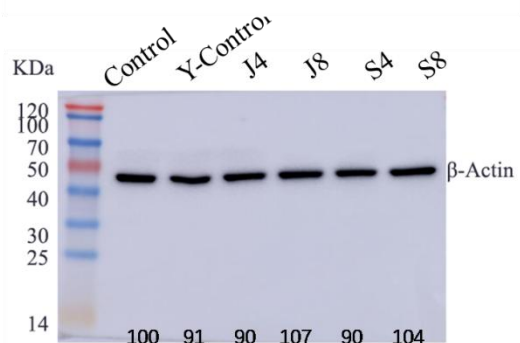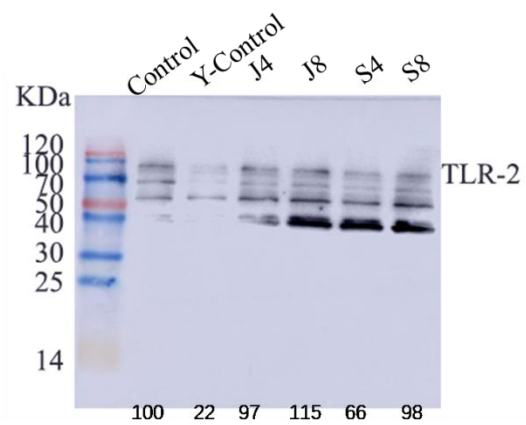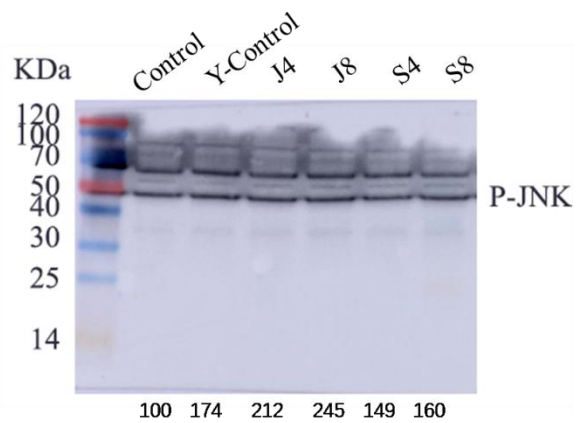

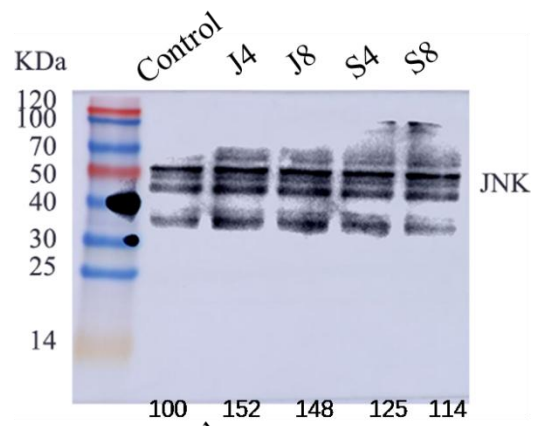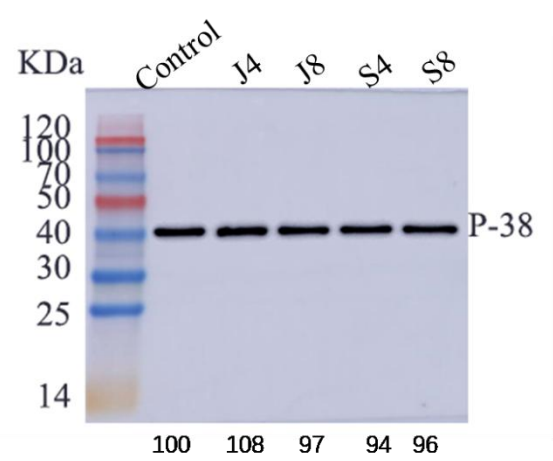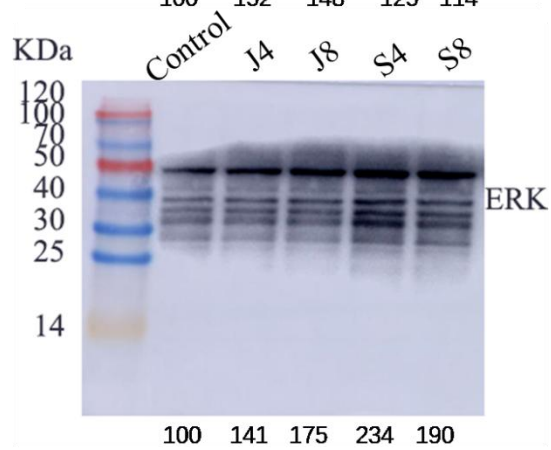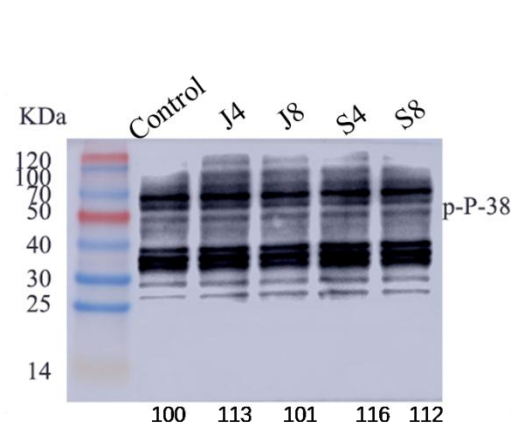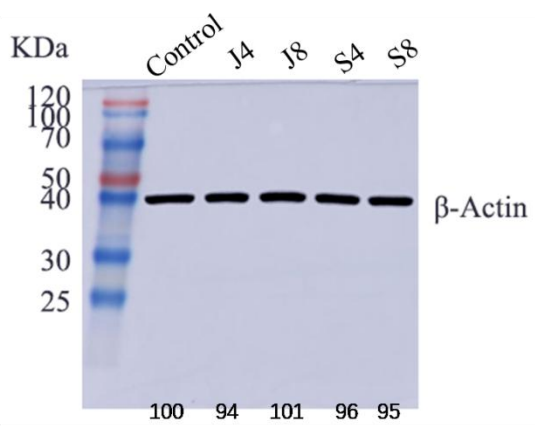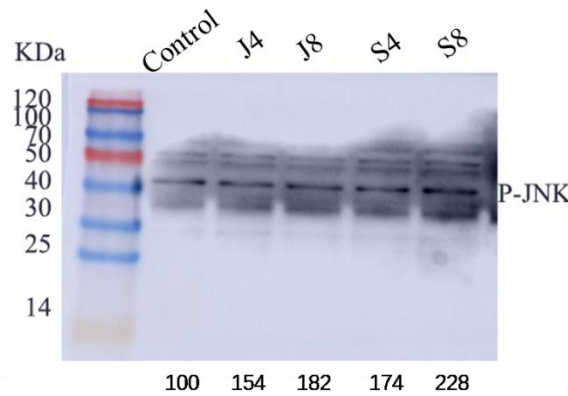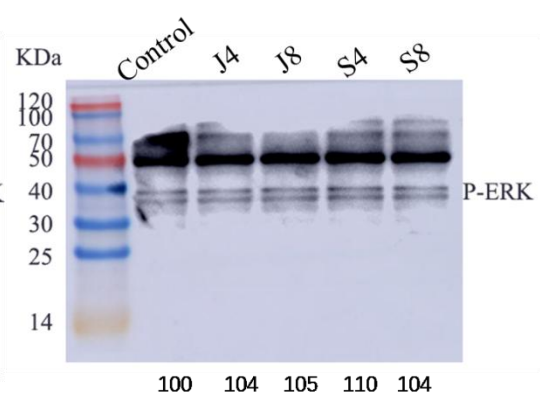

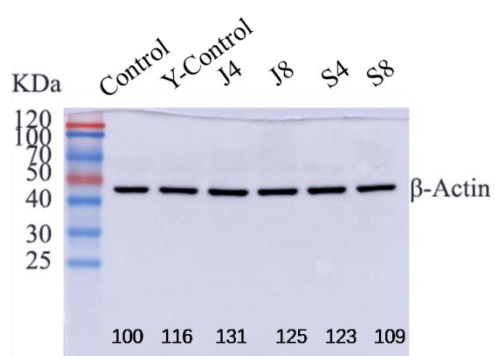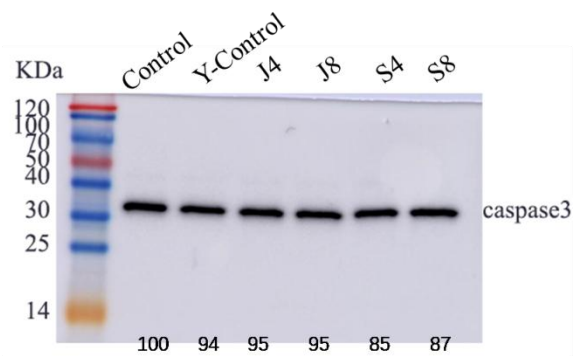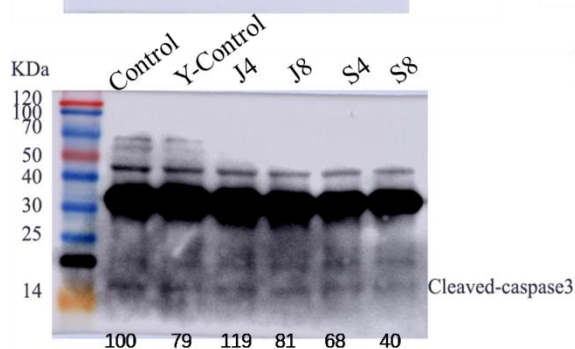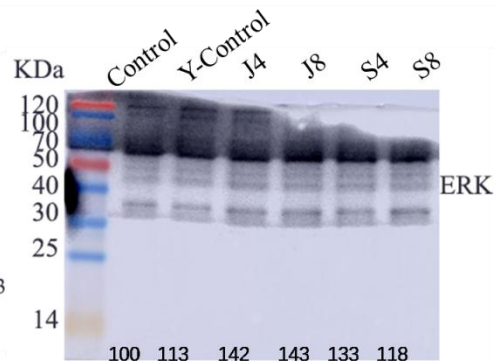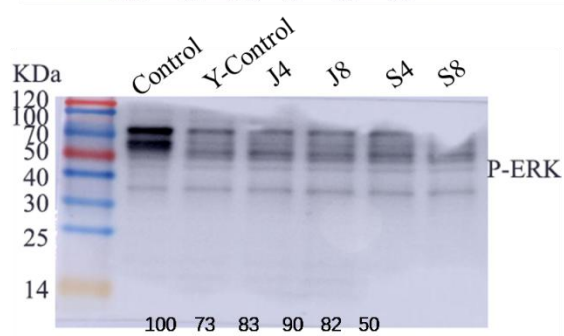

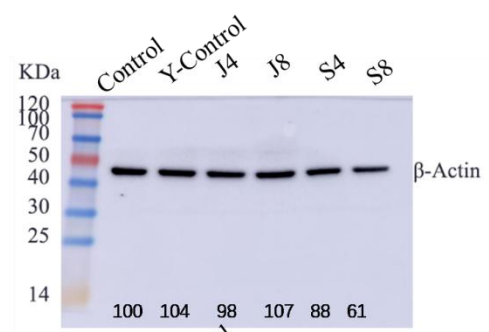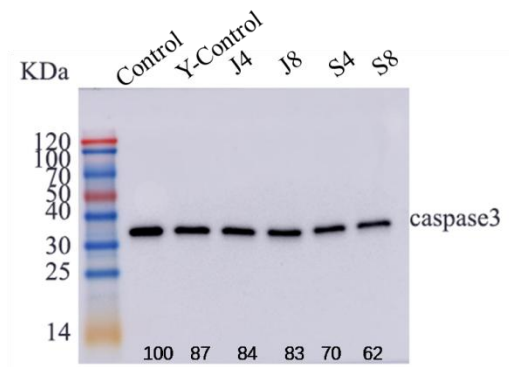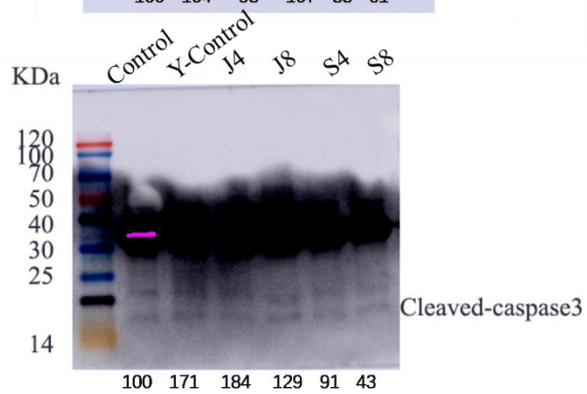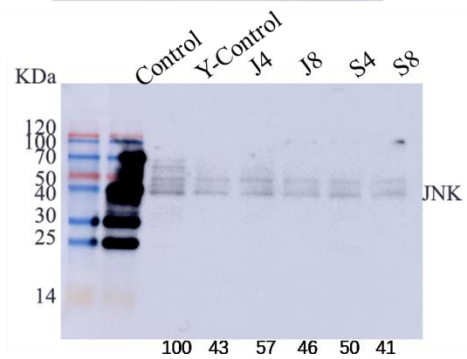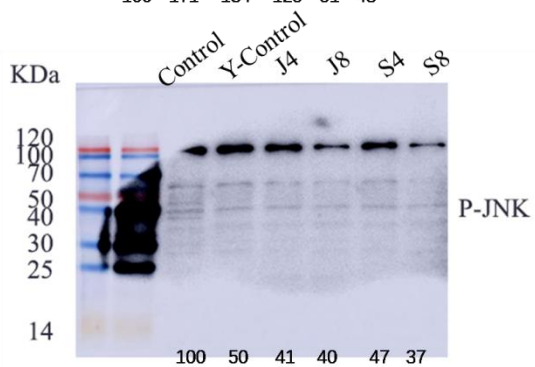

Supplement: Supplementary file 1 [file animals-13-03222-s001.zip › Supplemental Figure S2.pdf]
